# Is the presence of abnormal prion protein in the renal glomeruli of feline species presenting with FSE authentic?

**DOI:** 10.1186/1746-6148-6-41

**Published:** 2010-08-04

**Authors:** Stéphane Lezmi, Thierry GM Baron, Anna A Bencsik

**Affiliations:** 1 French agency for food, environmental and occupational health safety (Anses) Unité ATNC, 31, avenue Tony Garnier, 69364 Lyon cedex 07, France

## Abstract

In a recent paper written by Hilbe et al (BMC vet res, 2009), the nature and specificity of the prion protein deposition in the kidney of feline species affected with feline spongiform encephalopathy (FSE) were clearly considered doubtful. This article was brought to our attention because we published several years ago an immunodetection of abnormal prion protein in the kidney of a cheetah affected with FSE. At this time we were convinced of its specificity but without having all the possibilities to demonstrate it. As previously published by another group, the presence of abnormal prion protein in some renal glomeruli in domestic cats affected with FSE is indeed generally considered as doubtful mainly because of low intensity detected in this organ and because control kidneys from safe animals present also a weak prion immunolabelling. Here we come back on these studies and thought it would be helpful to relay our last data to the readers of BMC Vet res for future reference on this subject.

Here we come back on our material as it is possible to study and demonstrate the specificity of prion immunodetection using the PET-Blot method (Paraffin Embedded Tissue - Blot). It is admitted that this method allows detecting the Proteinase K (PK) resistant form of the abnormal prion protein (PrPres) without any confusion with unspecific immunoreaction. We re-analysed the kidney tissue versus adrenal gland and brain samples from the same cheetah affected with TSE using this PET-Blot method. The PET-Blot analysis revealed specific PrPres detection within the brain, adrenal gland and some glomeruli of the kidney, with a complete identicalness compared to our previous detection using immunohistochemistry. In conclusion, these new data enable us to confirm with assurance the presence of specific abnormal prion protein in the adrenal gland and in the kidney of the cheetah affected with FSE. It also emphasizes the usefulness for the re-examination of any available tissue blocks with the PET-Blot method as a sensitive complementary tool in case of doubtful PrP IHC results.

## Introduction

In a recent article relating to feline spongiform encephalopathy (FSE) in cats, the authors described the distribution of abnormal prion protein in different tissues detected using immunohistochemistry (IHC) method [1]. They described labelling of glomerular structures in the kidney of the affected cats with FSE as well as in control healthy cats using two anti-prion antibodies. Similar features were described in another study published earlier [2] and in which some control cats were also reported with traces of labelling in some glomerular structures of the kidney. With regard to these results, the presence of abnormal prion proteins in some renal glomeruli in feline species affected with FSE was considered doubtful by these authors and a non specific binding of antibody highly suspected.

Interestingly, we also reported in a previous work [3] the accumulation of deposits of prion protein in various organs of a cheetah affected with FSE. In that study we illustrated a dense accumulation of prion protein in some glomeruli of the kidney and also unexpected prion deposits in the cortex of the adrenal gland. Even if we used four different anti-prion antibodies to characterise these prion deposits, and also because we did not have the opportunity to test a kidney and adrenal gland from a healthy control cheetah at that time, the hypothesis of a non specific antibody binding could not be completely ruled out. Recently, we reported 2 other FSE cases in cheetahs, remarkable for the reason that they may represent a first probable case of maternal transmission of FSE [4]. However, in these cases, kidneys as well as adrenal glands were not available. To get rid of the question of the specificity of our observation in adrenal gland and kidneys described in our article of 2003, it is possible today to evaluate the specificity of the abnormal prion protein using the PET-blot method (Paraffin Embedded Tissue - Blot). This method offers indeed a greater specificity when compared to IHC [5], because during the PET-Blot process, a severe proteinase K treatment is applied that leads to complete digestion of cellular form of the prion as well as other proteins present in the tissues. Thus, only the PrP resistant form to PK digestion (the PrP form usually considered to be detected by western blot method) remains detectable, assuring the specificity of the detection. This method has already proved to be a highly sensitive and specific method to study different prion diseases in different species such as human TSEs [6-8], cattle BSE [5], sheep scrapie cases [9], experimental BSE and scrapie in mice [10], in hamster [11,12]. In addition it has been used with similar sensitivity to study PrPres in numerous peripheral tissues in humans [13] or such as placenta, muscle, skin in the sheep species [14-16]. In that context PET-Blot technique appeared as an excellent method for the detection of the regional distribution of the PK resistant PrP in the central and peripheral tissues from TSE affected humans and animals. We thus performed this PET-Blot analysis on the cerebellum, adrenal gland and kidney of the cheetah affected with FSE reported in our article published in 2003, in order to demonstrate the specificity of PrPres accumulation in these tissues.

## Materials and methods

The tissue samples were the same as those used in our previous study in which the tissue preparation is described in detail [3]. To visualize *in situ *the resistant form of abnormal PrP (PrP^res^), after digestion with high concentration of proteinase K (PK), the PET-blot method was also used as previously described [10]. Brain sections at the level of the obex from sheep affected or not with scrapie were used as positive and negative tissue control respectively. Concisely, paraffin sections of 5 μm were cut from cerebellum, adrenal gland and kidney paraffin blocks and collected onto 0.45 μm pore nitrocellulose membranes. The membranes were dewaxed and dried at room temperature (RT). After wetting with TBST (10 mM Tris HCl, pH 7.8; 100 mM NaCl; 0.05% Tween 20) enzymatic digestion was performed using 250 μg/ml PK in a buffer made of 10 mM Tris HCl, pH 7.8, 100 mM NaCl, 0.1% Brij 35, for 8 hours at 55°C, so that only PK resistant protein (PrP^res^) would be detectable. Membranes were treated with guanidine isothiocyanate (3 M, 10 min), then thoroughly washed in TBST. Immunodetection was performed after pre-incubation in blocking solution (skimmed milk diluted at 0.2% in TBST). The monoclonal antibody used was SAF84 (1/2500) for a night at room temperature. A phosphatase alkaline coupled anti-mouse antibody was used as secondary antibody (1/500, 37°C, 45 min). Before revelation, the pH was adjusted to 9 by incubating membranes in NTM buffer (100 mM Tris-HCl, pH 9.5, 100 mM NaCl, 50 mM MgCl2). Finally NBT/BCIP was used to visualize the reaction product (dark-blue deposits). PET-blot membranes were assessed using a stereo-microscope (Olympus, France) coupled to an image analysis workstation Explora Nova, La Rochelle, France. Immunohistochemical detection of the pathological form of the prion protein (PrP^d ^for disease-related) was performed using the SAF84 monoclonal antibody as published previously [4]. A peroxidase-labelled avidin-biotin complex was applied to amplify the signal. The presence of PrP^d ^was revealed using DAB intensified with chloride nickel giving dark deposits. The absence of detection of PrP^res ^in the obex of scrapie-free sheep combined with the presence of PrP^res ^seen in the obex of the scrapied sheep allowed to validate the PET-Blot procedure (data not shown).

## Results

### PET-Blot analysis reveals specific PrPres detection

**Figure 1:**
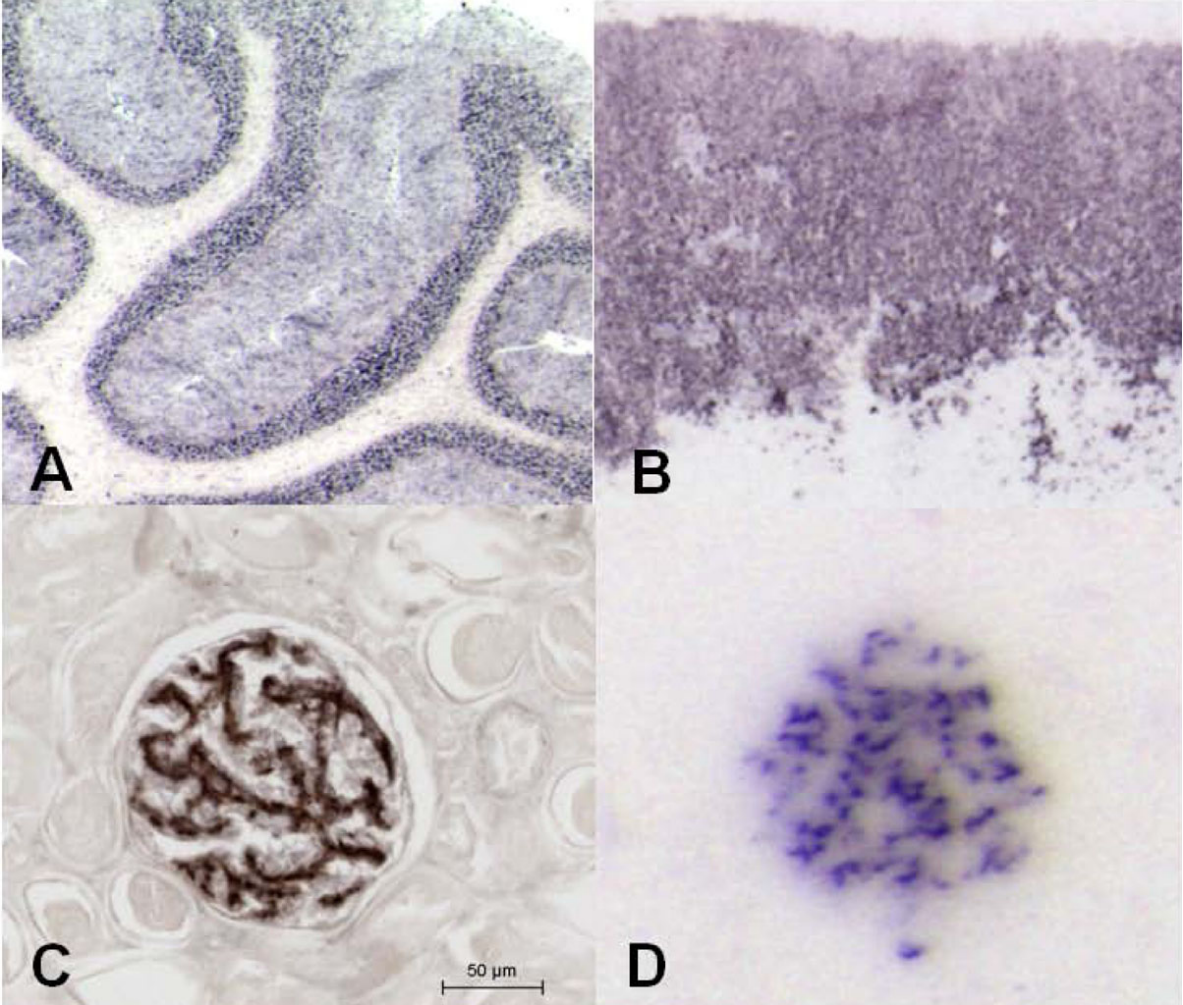
**PET-Blot analyses substantiate the specific PrPres detection in the kidney of cheetah with FSE**. PET-Blot analysis allows to reveal the presence of PrPres (dark deposits) in the cerebellum (A) as well as in the adrenal gland (zona fasciculata and reticularis) (B). In the kidney from a cheetah with FSE, using immunohistochemistry, PrPsc is detected in glomerules (C), the specificity of this intense labeling can be attested by the presence of the pathological form defined by its resistance to an enzymatic digestion (PrPres) detected using the PET-Blot method (D).

The abnormal form of prion protein characterized by its resistance to PK digestion was detected with high intensity in each sample analysed. When compared to our IHC results [3] we clearly identify on PET-blots an identical distribution of abnormal prion protein in the different layers of the cerebellum (figure 1 A) in the cheetah affected with FSE. As an internal control, the absence of PrPres deposition was also confirmed in the white matter of the cerebellum (figure 1 A). Similarly, an intense abnormal prion deposition was visualized in the cortex of the adrenal gland, in the zona fasciculata and zona reticularis as previously reported (figure 1B). In the kidney some glomeruli were also densely labelled (figure 1D) reproducing the glomerular labelling observed by IHC observable here as dark deposits of DAB intensified using chloride nickel (figure 1C).

## Discussion

These new results enable us to confirm confidently the presence of specific abnormal prion protein in the adrenal gland and in the kidney of the cheetah affected with FSE. This question is important because it becomes evidenced that urine may sustain transmission of certain forms of the transmissible spongiform encephalopathy (TSE) diseases, such as hamsters carrying infectious particles. More recently the kidney was found to accumulate abnormal PrP in other species too such as sheep [17,18], and the urinary secretion of pathological form of PrP is seriously considered [19,20]. Even if the origin of the production of this infectious prion particles are not yet clearly identified, the specific detection of PrPres within the glomeruli of the kidney of cheetah with FSE is in total accordance with this point.

The present study shows that the use of the PET-blot method would have been very interesting to test to clarify the presence of prion in the kidney of affected cats with FSE as well as in controls. This approach could have also a great interest in the characterization of the PrP staining observed in the adrenal gland. In the study of Hilbe et al [1], the adrenal gland staining was reported as being weak and only in the medulla, on the contrary to our finding in the cheetah FSE in which the staining was very weak in the medulla and particularly intense in the zona fasciculata and reticularis [3]. Thus it would be motivating to validate also the specificity of their labelling using the PET-Blot analysis, still because cats and cheetahs are not belonging to the same species, the tissue targeting of PrPsc deposition may be different.

Finally, the data reported here demonstrate the specificity of the detection of abnormal prion protein in the kidney as well as in adrenal gland in cheetah affected with FSE. It also emphasizes the need for the re-examination of available tissue blocks with the PET-Blot method as a sensitive complementary tool in case of doubtful PrP IHC results.

## Authors' contributions

AB conceived and designed the study, SL performed the PET-Blot experiments, AB and SL analyzed the data and wrote the paper. TB had overall responsibility as head unit. All co-authors have read and approved the final manuscript.
